# Pseudomonas pyocyanin stimulates IL-8 expression through MAPK and NF-κB pathways in differentiated U937 cells

**DOI:** 10.1186/1471-2180-14-26

**Published:** 2014-02-06

**Authors:** Wenshu Chai, Jia Zhang, Yan Duan, Dianzhu Pan, Wei Liu, Ying Li, Xue Yan, Baiyi Chen

**Affiliations:** 1 Department of Respiratory Diseases, the First Affiliated Hospital of Liaoning Medical University, Jinzhou, Liaoning, 121001, China; 2 Hebei Medical University, Shijiazhuang, Hebei 050000, China; 3 Department of Infectious Diseases, the First Affiliated Hospital of China Medical University, Shenyang, Liaoning 110001, China

**Keywords:** Pyocyanin, IL-8, U937 cell, p38, ERK, NF-κB

## Abstract

**Background:**

Pyocyanin (PCN), an extracellular product of *Pseudomonas aeruginosa* and a blue redox active secondary metabolite, plays an important role in invasive pulmonary infection. However, the detailed inflammatory response triggered by PCN infection in inflammatory cells (particularly macrophages), if present, remains to be clarified. To investigate the effects of PCN on macrophages, the ability of PCN to induce inflammation reaction and the signaling pathway for IL-8 release in PCN-induced differentiated U937 cells were examined.

**Results:**

It was found that PCN increased IL-8 release and mRNA expression in Phorbol 12-myristate 13-acetate (PMA) differentiated U937 cells in both a concentration- and time-dependent manner by reverse transcription-polymerase chain reaction (RT-PCR) and enzyme-linked immunosorbent assay (ELISA). P38 and ERK MAPKs were activated after 10 min of induction with PCN and their levels returned to baselines after 30 min by Western blotting. It was also found that within 10 min of PCN incubation, the level of p-I**-**κBα in the cytosol was increased, which returned to baseline level after 60 min. Meanwhile, the level of p-p65 was increased in the nuclear extract and cytosol, and maintained high in total cell lysates. The results were further confirmed by the observation that p38, ERK1/2 and NF-κB inhibitors inhibited PCN-induced NF-κB activation and attenuated PCN-induced IL-8 expression in U937 cells as a function of their concentrations. Moreover, it was shown that PCN induced oxidative stress in U937 cells and N-acetyl cysteine, an antioxidant, was able to inhibit PCN-induced IL-8 protein expression.

**Conclusions:**

It is concluded that PCN induces IL-8 secretion and mRNA expression in PMA-differentiated U937 cells in a concentration- and time- dependent manner. Furthermore, p38 and ERK MAPKs and NF-κΒ signaling pathways may be involved in the expression of IL-8 in PCN-incubated PMA-differentiated U937 cells.

## Background

*Pseudomonas aeruginosa* (*P*. aeruginosa), an opportunistic pathogen, causes infections associated with high incidences of morbidity and mortality in immunocompromised hosts. *P. aeruginosa* colonizes the lower respiratory tract in patients resulting in bronchiectasis, cystic fibrosis, and chronic obstructive pulmonary disease [1-3]. The pathogen has a broad host range, which produces a large number of extracellular products including elastase and alkaline protease, LasA protease, hemolysin, rhamnolipid, and pyocyanin (PCN). These extracellular products alter host cell function and may contribute to disease pathogenesis.

Among recognized virulence factors, the redox-active phenazine PCN, a blue redox active secondary metabolite, plays an important role in invasive pulmonary infection. Early studies have shown that PCN causes multiple effects on human cells, such as inhibition of cell respiration, ciliary function, epidermal cell growth, and prostacyclin release. Furthermore, PCN alters calcium homeostasis, causing damage to human cells. Recent studies have confirmed that PCN can alter the host’s immune response and increase IL-1 and TNF-α secretion induced by monocytes. PCN can also inhibit the body’s specific immune response to clear out pathogens, extend the time limit or prevent the infection of bacterial clearance, and increase secretion of inflammatory mediators in the body that can produce adverse reactions. Studies have also shown that PCN and its precursor, promethazine-1-carboxylic acid, change the host’s immune response by adjusting the RANTES [4] and IL-8 levels, and that in a variety of respiratory cell lines and primary cell cultures, PCN stimulation can cause the release of IL-8, IL-1 and IL-6 [5], accompanied by increased levels of IL-8 mRNA. PCN also acts in synergy with IL-1α, IL-1β and TNF-α to induce IL-8 expression in human airway epithelial cell lines [6-8]. In contrast to its effects on IL-8 expression, PCN inhibits cytokine-dependent expression of the monocyte/macrophage/T-cell chemokine RANTES. It is possible that the inhibition could cause inflammation of mononuclear macrophage and T cell influx to subside.

Alveolar macrophages are significant defense cells and inflammation regulatory cells which switch on multiplicity mediators of inflammation and cytokines and then cause acute lung injury. Although lung macrophages have the capacity to participate in the host response to *P. aeruginosa*, the role of alveolar macrophages in acute *P. aeruginosa* infection has not been clearly defined. The molecular mechanism by which these factors exert their effects is poorly understood. Human medullary system cell line U937 cells share characteristics with monoblasts and pedomonocytes. The human U937 promonocytic cell line was selected as the cell model since it is widely used to study the differentiation of promonocytes into monocyte-like cells [9-11]. Therefore, in this study, U937 cells were induced and differentiated into macrophages with phorbol 12-myristate 13-acetate (PMA) and used to study PCN effects on human macrophages.

*Pseudomonas* infections are characterized by a marked influx of polymorphonuclear cells (PMNs) (neutrophils) [12]. Increased release of IL-8, a potent neutrophil chemoattractant, in response to PCN may contribute to the marked infiltration of neutrophils and subsequent neutrophil-mediated tissue damage that are observed in *Pseudomonas*-associated lung diseases [7]. Previous studies by other investigators have identified a *Pseudomonas* secretory factor with the properties of PCN that increases IL-8 release by airway epithelial cells both *in vitro*[13] and *in vivo*[14]*.* Based on these studies, we examined the effect of PCN on IL-8 release *in vitro* using the human monocyte model (PMA-differentiated human promonocytic cell line U937) in synergy with inflammatory cytokines. The reasons for specific focus on IL-8 and nuclear factor-κB (NF-κB) pathway for IL-8 modulation are that IL-8 is an established enhancer of neutrophil function [5,6,8], while NF-κB is a transcription factor believed to play a key role in IL-8 expression [15].

Meanwhile, a number of studies have also shown that the mitogen-activated protein kinases (MAPKs, including ERK, JNK and p38) signal transduction pathways mediate a variety of stimulating factors-induced IL-8 expression [4,16-18]. NF-κB is a ubiquitous pleiotropic transcription factor. Studies have shown that NF-κΒ activation is a contributing factor for a variety of lung diseases and lung inflammation [19-21]. Pyrrolidine dithiocarbamate, a metal chelator and antioxidant, can inhibit the activation of NF-kB specifically by suppressing the release of the inhibitory subunit Ik-B from the latent cytoplasmic form of NF-kB. Recent studies have indicated that maximal IL-8 protein expression requires activation of NF-κB as well as MAPKs [17]. However, the precise relationship between NF-κB transactivation and MAPK activation remains unclear. In addition, few cellular pathways that are affected by PCN are known. Hence, the present study was designed to testify whether PCN can provoke the activation of macrophages, and whether NF-κB and MAPKs are involved in this possible process.

## Methods

### Chemicals and reagents

RPMI-1640, fetal bovine serum (FBS), and antibiotics were purchased from GIBCO BRL (Grand Island, NY). Phospho-specific p38 MAPK and p38, and phospho-specific ERK1/2 and ERK1/2 were from New EnglandBiolabs (Bevely, MA). Stocks of the selective p38 MAPK inhibitor SB203580, and stocks of the selective ERK1/2 inhibitor PD98059 were purchased from Calbio-chem-Behring (Za Jolla, CA). Phospho-NF-κB p65 (Ser276) antibody was purchased from Cell Signaling Technology (CST, Danvers, MA) and anti-p-IκB-α (Ser32) from Santa Cruz Biotechnology (Santa Cruz, CA) . IL-8 assay kit and TNF-α were purchased from R&D Systems (Minneapolis, MN). PMA was purchased from Merck Biosciences (San Diego, CA). PMS (phenazinem ethosulfate, molecular formula: C_14_H_14_N_2_O_4_S) was from AMRESCO (Solon, OH). NF-κB inhibitor PDTC, PCN, N-acetylcysteine, LDH, SOD,CAT, and MDA assay kits were purchased from Sigma Chemical Co. (St. Louis, MO). All other reagents, unless specified, were purchased from Sigma Chemical Co.

### Cell culture and differentiation

U937 cells were purchased from ATCC (American Type Culture Collection, Rockville, MD) and were cultured at 37°C in a humidified atmosphere with 5% CO_2_ in RPMI 1640 medium supplemented with 10% FCS and 50 μg/mL gentamicin, which itself was supplemented with 4.5 g/L glucose, 1 mM sodium pyruvate, and 10 mM HEPES. Cell culture was maintained at a density of 1 × 10^6^ cells/mL. All cell lines were diluted one day before each experiment. For differentiation into macrophages, U937 cells were treated with PMA (10 nM) and allowed to adhere for 48 h in a 5% CO_2_ tissue culture incubator at 37°C, after which they were washed and fed with PMA-free medium.

### Treatment with PCN and inhibitors

PMA-differentiated U937 cells were washed and afterwards different concentrations of PCN (5, 25, and 50 μM) were added into the medium and incubated for 24 h. Subsequently, the culture supernatant was collected and stored at -70°C. IL-8 concentration was measured by enzyme-linked immunosorbent assay (ELISA) assay. As a positive control, a separate group of PMA-differentiated U937 cells was stimulated with TNF-α and PCN. RNA was extracted afterwards, and IL-8 mRNA levels were determined. In some experiments, SB203580, PD98059 or PDTC was added into fresh medium of U937 cells at 60 min before PCN incubation.

### Thiazolyl blue tetrazolium bromide (MTT) assay

Cell viability was assessed using the MTT assay (Sigma) according to the manufacturer’s instructions.

### Measurement of IL-8

Cells were cultured in 24-well tissue culture plates until they reached 80-90% confluence. Cells were cultured in serum-free medium without growth factors and medium supplements for 24 h prior to treatment. The medium was harvested 24 h after treatment and stored at -20°C until assayed. IL-8 level was determined by ELISA according to the manufacturer’s instructions. The reproducibility, calculated as the coefficient of variation (CV), was 5.5%.

### Reverse transcription-polymerase chain reaction (RT-PCR)

Total RNA was extracted from the U937 cells as described by Chomczynski [22]. At the end of the incubation period, cells were washed with 1 mL ice-cold PBS and solubilized with 1 mL of trizol. RNA was treated with chloroform, centrifuged at 12000 × g for 15 min at 4°C and finally precipitated with ethanol. RNA was extracted and redissolved in diethylpyrocarbonate-treated water, and the OD at 260 nm was used to determine its concentration. To synthesize cDNA, 2.5 μg of RNA was resuspended in a 10 μL final volume of the reaction buffer and incubated for 30 min at 42°C. The reaction was stopped by denaturing the enzyme at 95°C for 5 min. Polymerase chain reaction was performed as follows. Ten microliters of the synthesized cDNA were added to 40 μL of PCR mixture containing 5 μL of 5 × PCR buffer, 1 μL of primers (GenBank accession IL-8 sense: 5′-AGATGTCAGTGCATAAAGACA-3′, antisense: 5′-TGAATTCTCAG CCCTCTTCAAAAA-3′, 201 bp; GenBank accession β-actin sense: 5′-GGCATGGGTCAGAAGGATY CC-3′, antisense: 5′-ATGTCACGCACGATTTCCCGC-3′, 501 bp) and 0.25 μL DNA polymerase. PCR conditions for IL-8 were 35 cycles of denaturation at 94°C for 45 s, annealing at 55.3°C for 45 s and extension at 72°C for 1 min. PCR conditions for β-actin were 35 cycles of denaturation at 94°C for 45 s, annealing at 59°C for 45 s and extension at 72°C for 1 min. Amplified PCR products were separated by electrophoresis on 1.5% agarose gel (UltraPure, Sigma) containing 0.05 μg/mL ethidium bromide. The mRNA expression was visualized using a Gel imaging system and analyzed using the molecular analyst software and was standardized by the β-actin housekeeping gene signal to correct any variability in gel loading. The ratio between the optical density of β-actin and the test gene was calculated to evaluate relative changes in the test gene.

### Western blotting

The cytoplasmic and nuclear extracts from differentiated U937 cells were prepared with NEPER Nuclear and Cytoplasmic Extraction Reagents (Pierce, Rockford, IL). Equal amounts (20 μg or 10 μg in the nuclear fraction) of protein extracts were electrophoresed on 8–10% SDS polyacrylamide gels and transferred onto polyvinylidene difluoride membranes. Rabbit anti-phospho-p65 (Ser276) and p-IκB-α (Ser32),rabbit anti- phospho-specific p38 MAPK and p38, rabbit anti-phospho-specific ERK1/2 and ERK1/2 were used to detect the presence of phospho-p65, phospho-specific p38 MAPK and p38; phosphor-specific ERK1/2 and ERK1/2, respectively. The scanned figures were visualized and quantified using Image J software.

### Statistical analysis

Data presented are representative of 3-5 independent experiments. Unless otherwise indicated, data were expressed as means ± S.D. Data were analyzed using one-way analysis of variance followed by LSD for multiple comparisons. Differences were considered significant if *p* < 0.05. All analyses were performed using SPSS 13.0 software.

## Results

### Induction of U937 cell differentiation by PMA

The U937 cells of a routine subculture are in the form of a single cell suspension. After 8 h of culture in the presence of 10 nM PMA, the cells began to transform from flat elongated suspension cells into irregular-shaped amoeba-like cells that developed pseudopodia extensions and adhered to the bottom of the container. After 48 h of cultivation, 85% of the cells were adherent growth. So far, differentiation of U937 cells by treatment with PMA has been accomplished.

### Cell viability assay

To assess the effect of PCN on cell viability, MTT assays were performed on cells incubated with a range of PCN concentrations (5-100 μM) after 24 h. Cell viability was not affected by PCN (5-75 μM). Loss of cell viability by 5-6% was observed at a PCN concentration of 100 μM (data not shown). Therefore, PCN concentrations ranging from 5 to 50 μM was used in the subsequent experiments.

### Effect of PCN on IL-8 mRNA

**Figure 1:**
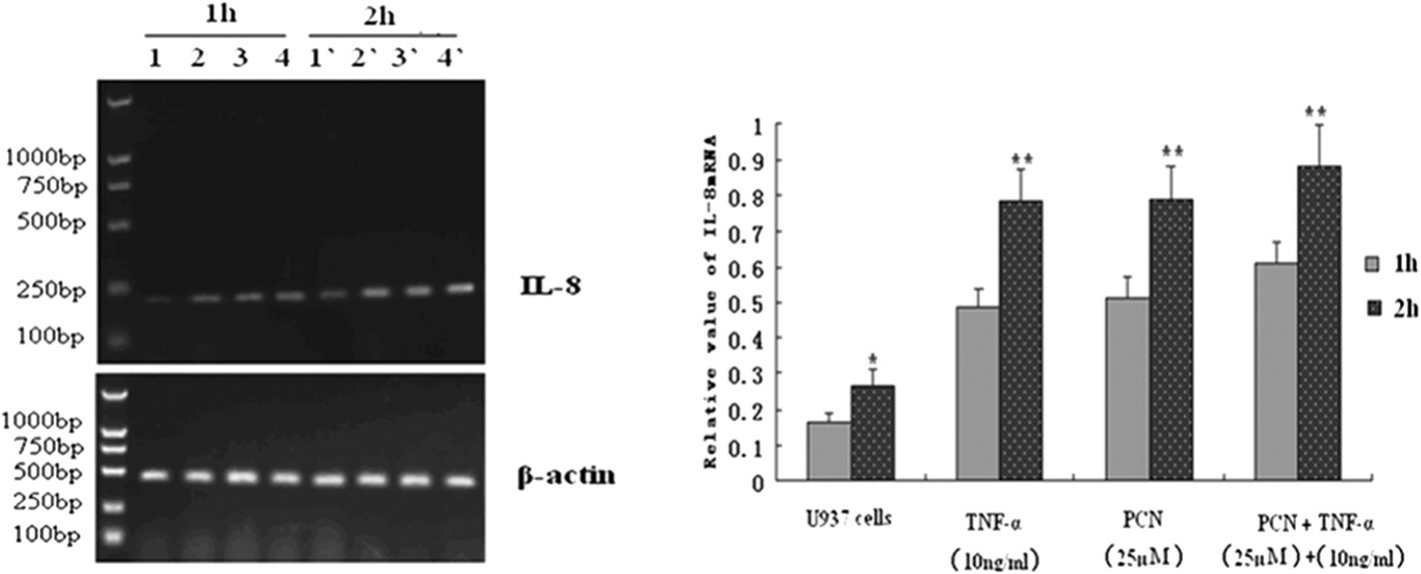
**The expression of IL-8 mRNA in PMA-differentiated U937 cells.** RNA was extracted from PMA-differentiated U937 cells after various treatments (TNF-α at 10 ng/mL and PCN at 25 μM and 25 μM PCN combined 10 ng/mL TNF-α respectively) with the indicated time periods, and analyzed by RT-PCR with specific primers for IL-8. Results shown are representative of three separate experiments. Expression of IL-8 mRNA was quantified by densitometry, and standardized by the β-actin level. *p < 0.05, **p < 0.01 compared with the level at 1 h or 2 h. PMA: phorbol 12-myristate 13-acetate.

In these studies, TNF-α was used as a positive control to further explore the expression of IL-8 mRNA induced by PCN. After treatments with TNF-α (10 ng/mL) or PCN (25 μM) alone or their combination for the indicated periods, IL-8 mRNA levels were analyzed by RT-PCR with its specific primers. PCN-mediated induction of IL-8 mRNA in differentiated U937 cells was detectable at any time point studied. TNF-α alone induced IL-8 mRNA in a time-dependent manner, which peaked at 2 h, and stimulated IL-8 release in a concentration-dependent manner after 24 hours of incubation (Figure 1). The medium alone produced trace amounts of IL-8. Treatment with PCN plus TNF-α slightly increased IL-8 mRNA expression. This difference, however, was not statistically significant (*p* > 0.05).

### Induction of IL-8 release by PCN in PMA-differentiated U937 cells

**Figure 2:**
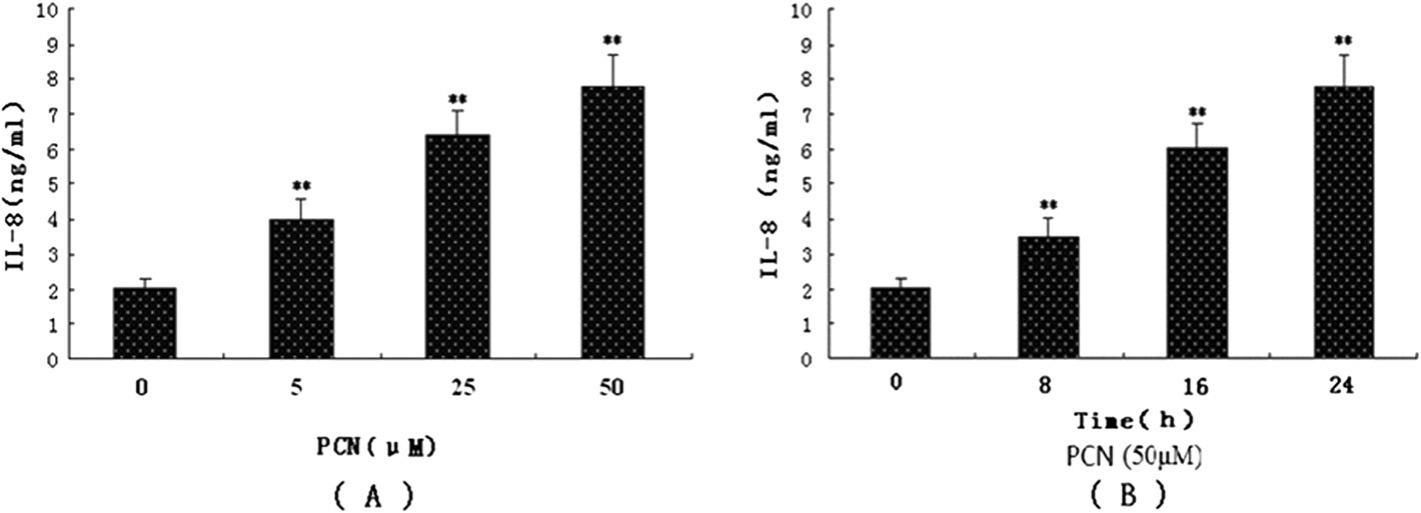
**PCN increases IL-8 release in PMA-differentiated U937 cells. (A)** Different concentrations of PCN (5 μM, 25 μM, or 50 μM) were added to the cell cultures for 24 h. Supernatants were harvested for measuring IL-8 secretion by ELISA. **(B)** A fixed concentration of PCN (50 μM) was added to the cell cultures for 8, 16 or 24 h. Supernatants were harvested for measuring IL-8 level by ELISA. Values represented are the mean ± SD of four independent experiments in triplicate. **p < 0.01 compared with PMA-differentiated U937 cells. PMA: phorbol 12-myristate 13-acetate.

Previous studies have identified that PCN stimulates IL-8 production by lung macrophage cells [23] and surface epithelial cells [8,14,24]. Based on the physical properties of PCN, we hypothesized that it was able to stimulate differentiated U937 cells to produce IL-8. To test this hypothesis, we exposed differentiated human U937 cells to purified PCN and measured its effects on the release of IL-8. After 24 hours of incubation with different concentrations of PCN (5 μM, 25 μM, or 50 μM) in PMA-differentiated U937cells, the supernatants were collected and IL-8 release detected by ELISA. The results showed that PCN increased IL-8 release in differentiated U937 cells in a concentration-dependent manner. An increase in IL-8 release was observed with PCN concentration at as low as 5 μM and the concentration of 50 μM produced the strongest stimulation as to the cellular response (Figure 2A and B). The increase in IL-8 above control levels was observed at as early as 8 h after PCN (50 μM) addition, and these levels continued to increase between 24 h and 48 h (data not shown). Longer periods of incubation were not tested.

### The oxidative effect of PCN on differentiated U937 cells

A previous study has shown that PCN induces a concentration-dependent loss of cellular glutathione (GSH), an important cellular antioxidant, up to 50% in the tissues infected by P. aeruginosa [25]. N-acetyl cysteine (NAC) is the precursor of GSH. So we hypothesized that NAC may play a protective role in cells exposed to PCN. Thus, different concentrations of PCN (5, 25, and 50 μM) were added into differentiated U937 cells, and the supernatants were collected after 24 hours. We then detected the leakage of LDH, the content of MDA, and the activities of SOD and CAT using their respective detection kits. Results showed that the leakage of LDH and the content of MDA increased and the activity of SOD and CAT decreased, all in a dose-dependent manner. There was a significant difference among the experimental groups (p < 0. 01) (Table 1). These results indicated PCN can induce oxidative damage.

**Table 1:** The oxidative effect of pyocyanin on differentiated U937 cells

**(**$\bar{x}$** ± s n=3)**				
**Group**	**LDH (U · L**^**-1**^**)**	**MDA (mmol · L**^**-1**^**)**	**SOD (Eu · mL**^**-1**^**)**	**CAT (Eu.mL**^**-1**^**)**
C_0_	301 ± 48	0.91 ± 0.07	5.99 ± 0.96	1.86 ± 0.21
C_1_	521 ± 48**	2.01 ± 0.23**	4.66 ± 0.75*	1.27 ± 0.18*
C_2_	590 ± 52**	2.93 ± 0.19**	3.86 ± 0.62**	1.01 ± 0.14**
C_3_	668 ± 76**	3.85 ± 0.25**	3.12 ± 0.41**	0.62 ± 0.11**

Notice: C_0_: Control group; C_1_: PCN (5 μM); C_2_: PCN (25 μM); C_3_: PCN (50 μM).

*P < 0.05, compared with control; **P < 0.01, compared with control.

### Effects of MAPK inhibitors on PCN-induced IL-8 release

**Figure 3:**
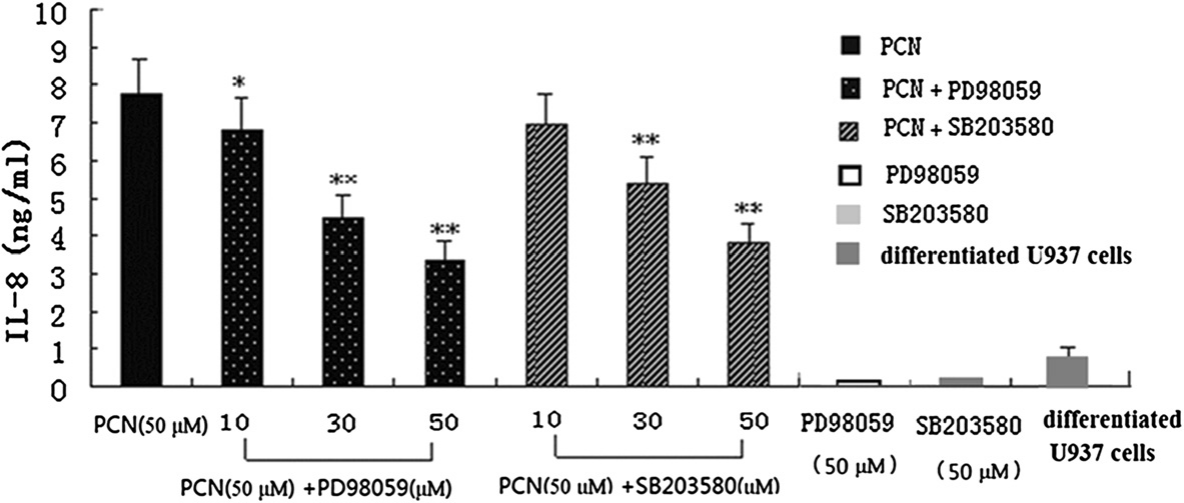
**MAPK inhibitors attenuate PCN-induced IL-8 release.** Different concentrations of the ERK or P38MAPK blockers (PD98059 at 10, 30, or 50 μM or SB203580 at 10, 30, or 50 μM) were added into fresh medium of PMA-differentiated U937 cells 60 min before PCN was added. Cells were exposed to PCN (50 μM) for 24 h. Supernatants were harvested for measuring IL-8 by ELISA. **p < 0.01 compared with PMA-differentiated U937 cells. MAPK: mitogen-activated protein kinase; ERK: extracellular signal-regulated kinase; PMA: phorbol 12-myristate 13-acetate.

A number of studies show that the MAPK signal transduction pathways mediate IL-8 expressions induced by a variety of stimulating factors [26]. We therefore went on to explore the possibility that PCN may induce U937 cells to express IL-8 through MAPK signaling. In some experiments, different concentrations of the ERK and P38 MAPK blockers (PD98059 at 10, 30, or 50 μM and SB203580 at 10, 30, or 50 μM, respectively) were added into the fresh medium of U937 cells 60 min before PCN addition. After 24 hours, the supernatants were collected and IL-8 concentrations were detected by ELISA. The results showed that PD98059 and SB203580 significantly decreased the secretion of IL-8, and as either substance’s concentration increased, IL-8 secretion decreased, indicating that PCN may stimulate U937 cells to express IL-8 by both MAPK signaling pathways (Figure 3).

### Effects of NF-κB inhibitor on PCN-induced IL-8 release

**Figure 4:**
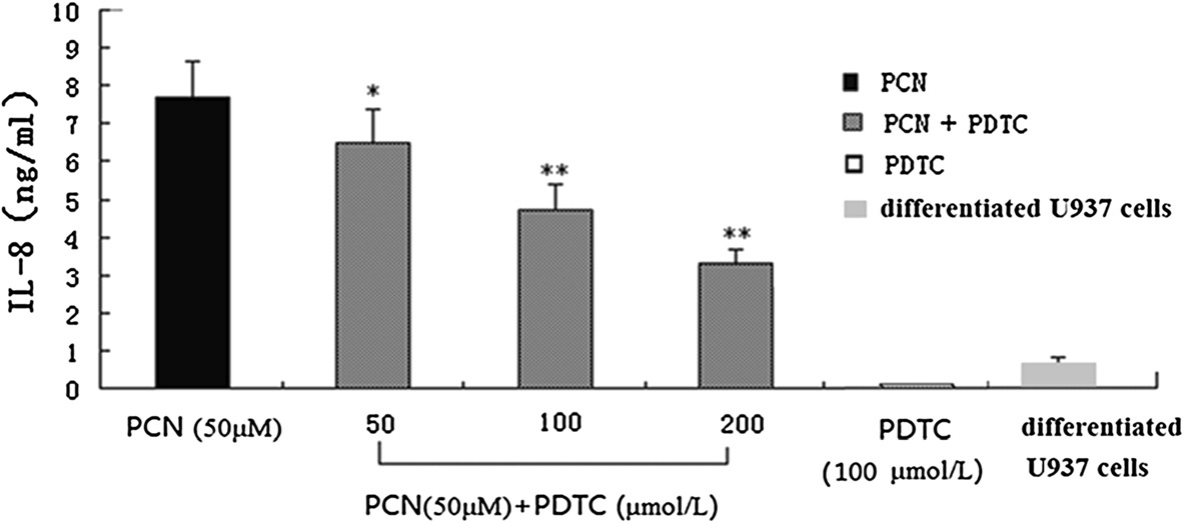
**NF-κB inhibitor reduces PCN-induced IL-8 release.** Different concentrations of NF-κB blocker (PDTC at 50, 100, or 200 μmol/L) were added into fresh medium of PMA-differentiated U937 cells 60 min before PCN was added. Cells were exposed to a fixed concentration of PCN (50 μM) for 24 h. Supernatants were harvested for measuring IL-8 by ELISA. *p < 0.05, **p < 0.01 compared with the PCN group. PMA: phorbol 12-myristate 13-acetate.

To further investigate whether NF-κB is involved in PCN-induced IL-8 production, different concentrations of NF-κB blockers (PDTC at 50, 100, or 200 μmol/L) were added into fresh medium of PMA-differentiated U937 cells 60 min before PCN was added. After 24 hours of further incubation, the supernatants were collected and IL-8 concentrations were detected. Results showed that PDTC significantly decreased the secretion of IL-8, and with increasing concentrations PDTC, IL-8 secretion decreased, although in the presence of high concentrations of PCN, indicating that the PCN may stimulate PMA-differentiated U937 cells to express IL-8 by NF-κB signaling pathway (Figure 4).

### Effect of antioxidant on PCN-induced IL-8 release

**Figure 5:**
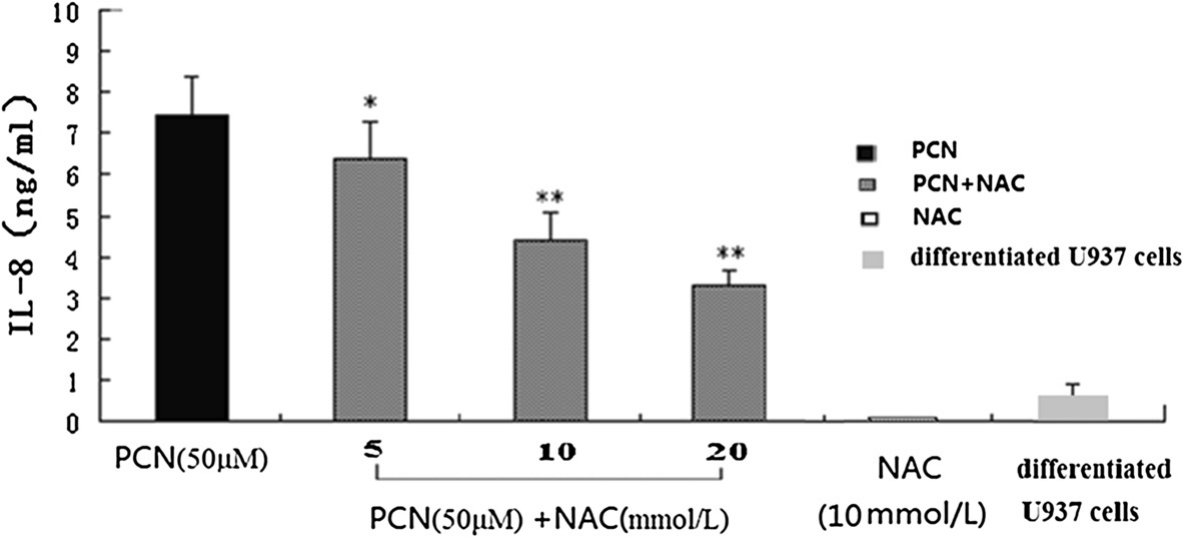
**Antioxidant can inhibit PCN-induced IL-8 release.** Different concentrations of N-acetyl cysteine (NAC) (5, 10 or 20 mM) were added into fresh medium of PMA-differentiated U937 cells for 60 min before PCN was added. After 24 h, supernatants were collected and IL-8 concentrations were detected by ELISA. *p <0.05, **p < 0.01 compared with the PCN groups. PMA: phorbol 12-myristate 13-acetate.

To further authenticate whether oxidative stress was involved in PCN-induced IL-8 production and protective role of NAC in cells exposed to PCN, different concentrations of NAC (5, 10, or 20 mmol/L) were added into fresh medium of PMA-differentiated U937 cells 60 min before PCN administration. After 24 hours of further incubation, supernatants were collected and IL-8 concentrations were measured. The results showed that NAC significantly decrease the secretion of IL-8, indicating a pivotal role for oxidative stress in PCN-induced IL-8 expression in PMA-differentiated U937 cells (Figure 5).

### Effects of MAPK and NF-κB inhibitors on PCN-induced IL-8 mRNA

**Figure 6:**
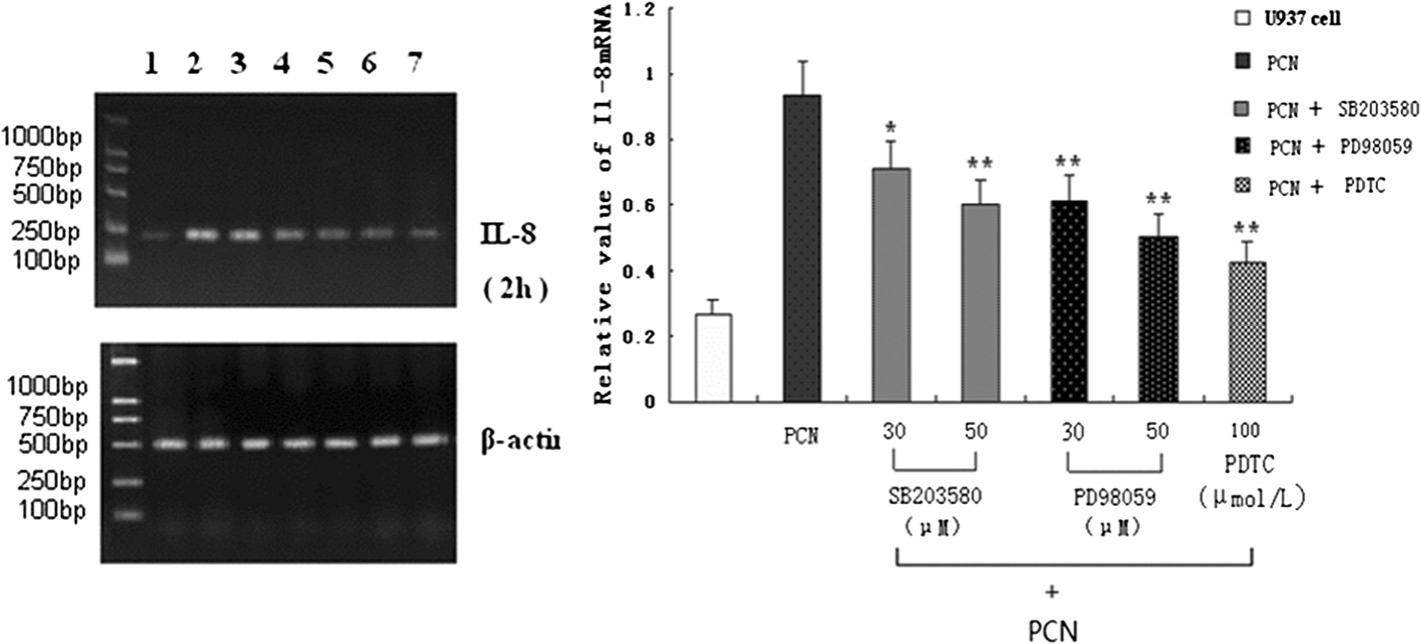
**MAPKs and NF-κB inhibitors can attenuate PCN-induced IL-8 mRNA.** PMA-differentiated U937 cells were pretreated for 60 min with SB203580 (30 μM or 50 μM), PD98059 (30 μM or 50 μM) or PDTC (200 μM) and then stimulated for 2 h with 50 μM PCN. Inhibitors were present throughout. RNA was then isolated, and levels of mRNA were determined. Expression of IL-8 mRNA was quantified by densitometry and standardized by β-actin. *p < 0.05, **p < 0.01 compared with PCN. MAPK: mitogen-activated protein kinase; PMA: phorbol 12-myristate 13-acetate.

To determine whether activation of MAPK and NF-κB mediates the PCN-dependent increase in IL-8 mRNA, we tested the effects of several MAPK and NF-κB inhibitors: SB203580 (a p38 inhibitor, 30 μM or 50 μM) and PD98059 (an ERK1/2 inhibitor, 30 μM or 50 μM) or PDTC (an NF-κB inhibitor, 200 μM). For these experiments, cells were pretreated for 60 min with SB203580, PD98059, or PDTC and then stimulated for 2 h with 50 μM PCN. The respective inhibitor was present throughout the experiments. RNA was then isolated and levels of mRNA were determined as described in materials and methods. The results showed that all blockers used can reduce the expression of IL-8 mRNA (Figure 6).

### PCN increases phosphorylation of p38 and ERK1/2 MAPKs

**Figure 7:**
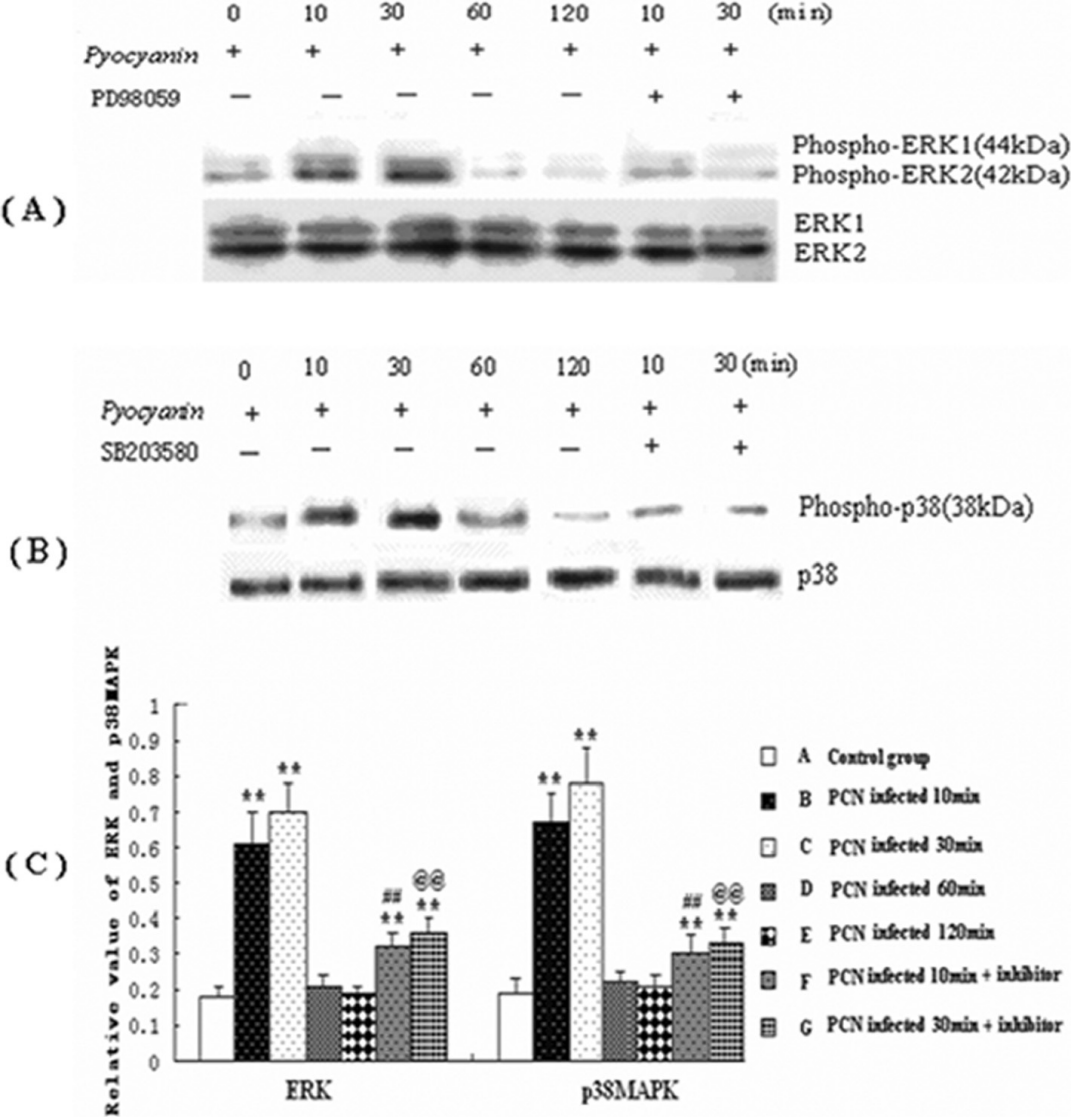
**The expression of phosphorylated and total MAPK proteins in PMA-differentiated U937 cells.** PMA-differentiated U937 cells were stimulated with PCN (50 μM) for the indicated time periods with or without pretreatment by MAPK inhibitor SB 203580 (30 μM) or PD98059 (30 μM ) for 1 h. **(A and B)** The expressions of phospho-ERK or ERK **(A)** and phospho-p38 or p38 **(B)**. **(C)** The expression of phosphorylated and total p38 and ERK proteins in U937 cells. Representative data of three independent experiments are shown. **p < 0.01 compared with the A group; MAPK: mitogen-activated protein kinase; ERK: extracellular signal-regulated kinase; PMA: phorbol 12-myristate 13-acetate.

To gain direct insights into PCN effect on MAPK activation, we then used PCN (50 μM) to stimulate U937 cells with or without pretreatment with MAPK inhibitors (SB 20358 or PD98059, both at 30 μM) for 1 h. Cellular protein was collected at 0, 10, 30, 60, and 120 min after PCN treatment. The kinetics of p38 and ERK activation after induction were assessed by Western blotting using antibodies that specifically recognize the phosphorylated forms of p38 and ERK MAPKs. Active p38 was detected in PMA-differentiated U937 cells induced by PCN, but the activation was transient, appearing at 10 and 30 min and returned to baseline level after another 30 min. Exposure of PMA-differentiated U937 cells to PCN for 30 min reduced activation of ERK1/2. After 30 min of induction, activation of ERK1/2 began to recover but then its activation was down-regulated in a time-dependent manner, while the total ERK, p38MAPK levels remained almost unchanged throughout the experimental period (Figure 7).

### PCN stimulated U937 cells to activate NF-κB signaling pathway

**Figure 8:**
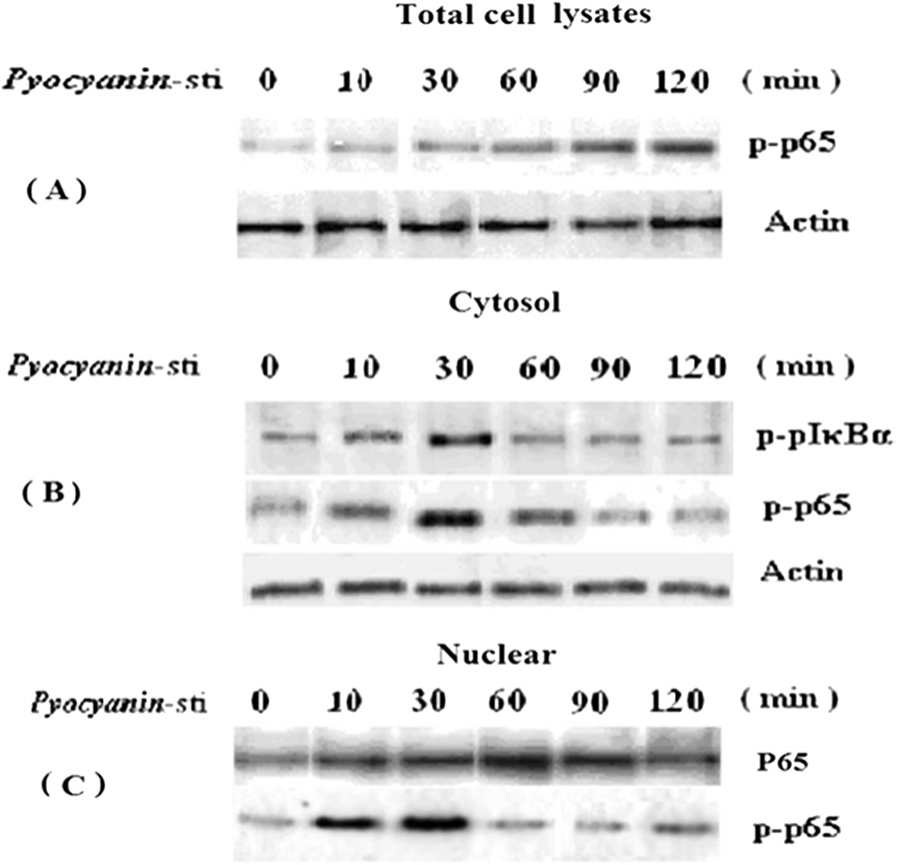
**PCN activates NF-κB signaling pathway.** Differentiated U937 cells were stimulated with PCN (50 μM). At 0, 10, 30, 60, 90 and 120 min, cell proteins were collected. Cytosolic or nuclear protein was extracted, and Western blotting was performed to detect NF-κB p65 protein translocation. Levels of phospho-p65 (Ser276) in whole cell lysates **(A)**, cytosol **(B)** or nuclear contents **(C)** were examined by Western blot analysis with the respective antibodies. Levels of p65 was also determined in nuclear fractions. β-actin was used as a control for equal loading. Data are the summary of averaged relative density units measured in 3 independent experiments. *p < 0.05 compared with control.

Activation of the NF-κB signaling pathway is frequently involved in the regulation of many immune response and inflammatory genes [27]. To determine whether PCN affects NF-κB signaling pathway, we examined the effect of PCN treatment on a series of molecular events that leads to NF-κB activation, including degradation of I-κBα protein, translocation of p65 to the nucleus, and the phosphorylation of p65. We used PCN (50 μM) to stimulate PMA-differentiated U937 cells. At 0, 10, 30, 60, 90, and 120 min, cell proteins were collected and NF-κB p65 protein translocation was detected by Western blotting. As shown in Figure 8, within 10 min after addition of PCN, the level of p-I**-**κBα in the cytosol was increased, which returned to baseline level after 60 min. We further investigated the change in nuclear localization of p65 protein. Within 10 min after addition of PCN, the level of p-p65 in total cell lysate and cytosol was increased. There was also an increase in the levels of p-p65 in the nuclear extract, as evidenced by high levels of p-p65 which persisted in total cell lysates (Figure 8). These results suggest that PCN induces degradation of I-κBα and subsequent translocation of NF-κB to the nucleus.

### Effects of MAPK inhibitors on PCN-induced NF-κB signaling activation

**Figure 9:**
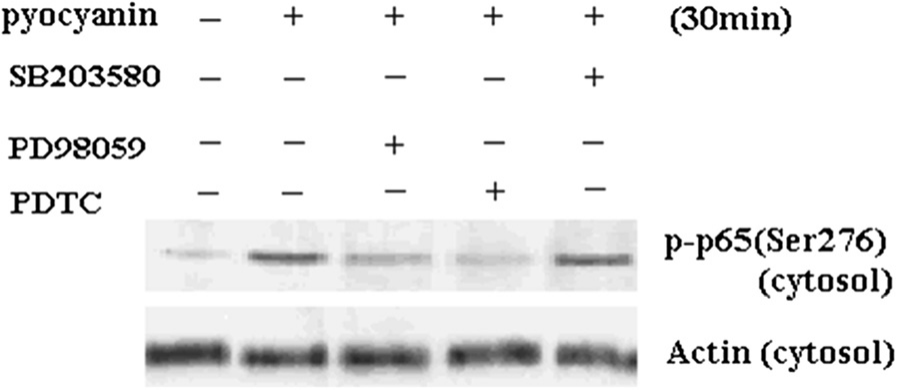
**Effects of MAPK inhibitors on PCN-induced NF-κB signaling pathway.** U937 cells were stimulated with PCN at 50 μM for the time periods indicated with or without pretreatment by MAPK and NF-κB inhibitors: SB 203580 (50 μM), PD98059 (50 μM) and PDTC (200 μM) for 1 h. Cell proteins were then collected and NF-κB p65 protein expression was detected with Western blotting.

To determine whether MAPKs mediate PCN-activated NF-κB signaling pathway, we used PCN (50 μM) to stimulate U937 cells with or without pretreatment with MAPK and NF-κB inhibitors: SB 203580 (50 μM), PD98059 (50 μM) and PDTC 200 μM for 1 h. Cell proteins were collected at 30 min and NF-κB p65 protein translocation was detected by Western blotting. The results showed that there was abundant cytosol distribution of NF-κB p65 before stimulation. All the indicated blockers were able to reduce the localization of NF-κB p65 in the cytosol (Figure 9). These data suggest that SB203580 and PD98059 can effectively inhibit PCN-induced NF-κB signaling activation. Therefore, it could be concluded that the activation of p38 and ERK MAPKs are signaling events that lie upstream of NF-κB activation.

## Discussion

The National Nosocomial Infection Surveillance indicates that *P. aeruginosa* is the second most common cause of nosocomial pneumonia after *Staphylococcus aureus*[28]. Ventilator-associated pneumonia (VAP) caused by *P. aeruginosa* is a severe complication of intensive care, with mortality rates of 34 to 48% [28-30]. Therefore, it is critical to study the pathogenesis of *P. aeruginosa*. In recent years, with the development of technologies such as the gene chip and the protein chip, and the clarification of the genome sequence of the *P. aeruginosa* strain, it has been found that many elements such as pro-inflammatory cytokines, antimicrobial peptides, complements and epithelial cell receptors and their signal transduction systems (TLR2, 4, 5, CFTR, GM1, and its downstream NF-κB) participate in host defense and immune response induced by *P. aeruginosa*. It has also been found that *P. aeruginosa* components (flagella and pili) and virulence factors (such as the density-sensing system, type secretion system, toxins, alginate and cell toxin) play important roles in the pathogenesis [2,16]. Among them, most *P. aeruginosa* strains secrete PCN (N-methyl-1-hydroxyphenazine), the pigment that gives blue-green color to the bacterial colonies [4]. High concentrations of PCN are detected in pulmonary secretions of patients with cystic fibrosis, where it triggers inflammation, disrupts the bronchial epithelium and impairs ciliary function. PCN also interferes with the antioxidant defenses in the lung and facilitates oxidative damage to the lung epithelium [31-35]. PCN has been detected at concentrations as high as 100 μM in pulmonary secretions from patients with *P. aeruginosa*-associated airway disease [36], and its production is increased when the organism is in the biofilm form [4,37]. Therefore, PCN plays an important role in acute and chronic invasive infections.

*Pseudomonas* infections are characterized by a marked influx of polymorphonuclear cells (PMNs) (neutrophils) [12]. Activated PMNs release a variety of oxidants and proteases that may contribute to the tissue injury that is observed in *Pseudomonas*-infected airways [12,38]. Little is known about the stimuli that are responsible for the influx and activation of PMNs into the presence of this bacterium. IL-8 is the major PMN chemoattractant responsible for PMN influx and activation in a variety of disease states and thus likely plays an important role in *P. aeruginosa* infections as well. It has been found that culture supernatants and various purified secretion factors of *P. aeruginosa* such as pili protein, flagellin, self-sensing materials, elastase, PCN and nitrite reductase [4,13,36,39,40] increase IL-8 secretion in airway epithelial cells, primary bronchial gland epithelial cells both *in vivo* and *in vitro*[40]. It was found that with NF-κB activation, rapid and sustained IL-8 mRNA expression was induced [37].

Recent studies have also further confirmed that in a variety of respiratory cell lines and primary cultures of cells, PCN stimulation can cause the release of IL-8, accompanied by increased IL-8 mRNA expression. PCN also acts in synergy with IL-1α, IL-1β and TNF-α to induce IL-8 expression [5,6,8]. After PCN was injected into animals and the respiratory tracts, bronchial lavage fluid and neutrophil (PMN) levels were increased significantly [41]. However, there are few reports on PCN effect on macrophages.

Our experimental results show that PCN induced expression of IL-8 in PMA-differentiated U937 cells, as well as IL-8 protein secretion and mRNA expression in a concentration- and time- dependent manner. It is also found that PCN synergizes with TNF-α to induce the expression of IL-8 in PMA-differentiated U937 cells. So far, most studies only observe the pro-inflammatory effects of the *P. aeruginosa* bacterial products on epithelial cells and macrophages, and their effects on U937 cells are less than well defined. The present study extends these findings by demonstrating that MAPKs and NF-κB signalings lie behind PCN-induced IL-8 production in differentiated U937 cells.

The MAPK family has an important role in signal transduction, and the pathway is activated by a variety of stimuli such as growth factors and cellular stresses [42,43]. Activated MAPKs can regulate the expression of inflammatory cytokines. In mammalian cells, it has been found that there are at least three major MAP kinase (MAPK) pathways including the extracellular signal-regulated kinase pathway (ERK), c-Jun N-terminal kinase/stress-activated protein kinase pathway (JNK), and the P38 MAPK pathway. A unique feature of the MAPKs is that they become activated after phosphorylation of both their tyrosine and threonine amino acids [44]. They are different activated extracellular signals that produce different biological effects. It has been found that MAPKs can modulate the expression of IL-8 in human peripheral blood mononuclear cells, granulocytes, mast cells, intestinal epithelial cells, and pulmonary vascular endothelial cells and that the use of P38 inhibitors can reduce the IL-8 mRNA and protein expression [19,23,41,45].

We used PCN to stimulate PMA-differentiated U937 cells and found that PCN could induce ERK and P38 MAPK protein phosphorylation, thus indicating the possible participation of ERK and p38 MAPK pathways in the regulation of IL-8. Our further investigation using MAPK pathway inhibitors PD98059 and SB203580 demonstrated that they may partially inhibit the phosphorylation and reduce IL-8 synthesis induced by PCN in a concentration-dependent manner, indicating that PCN may stimulate PMA-differentiated U937 cells to express cytokine IL-8 by MAPK signaling pathways.

NF-κB is a ubiquitous pleiotropic transcription factor, and studies have shown that NF-κΒ activation is critically involved in a variety of lung diseases and lung inflammation [19-21]. NF-κB activation can regulate a series of lung gene expression related to inflammatory and immune responses: pro-inflammatory cytokines such as TNF-α, IL-1β, chemokines MCP-1, IL-8, and many other molecules. Therefore, its activity is closely related with acute lung injury (ALI) and acute respiratory distress syndrome (ARDS) [46]. In most cell types, NF-kB is retained usually in the cytoplasm of the unstimulated cells by I-kBα family proteins. Upon stimulation, the I-kBα kinase complex is activated, resulting in the phosphorylation of I-kBs [47,48] The phosphorylated IkBs are ubiquitinated and subsequently degraded, which will release the transcription factor NF-kB [36,37]. In this study, we also found that PCN stimulation was associated with a significant increase in the level of phosphorylated I-kBα in total cell lysates. We further demonstrated that I-kBα decrease was accompanied by increased nuclear localization of p65 protein. These results suggest that PCN induces degradation of I**-**κBα and the subsequent translocation of NF-κB to the nucleus. The results also showed that different blockers (SB203580,PD98059 and PDTC) can reduce the expression of NF-κB p65 expression in cytosol and IL-8 expression, indicating that PCN may stimulate PMA-differentiated U937 cells to express cytokines IL-8 by MAPK and NF-κB signaling pathways.

Acute and chronic pulmonary infection with *P. aeruginosa* is associated with an intense neutrophil inflammatory response that contributes to lung injury [49]. A previous study has shown that PCN enhances airway epithelial cell release of IL-8 [4], a neutrophil chemokine whose production is regulated by oxidant-sensitive transcription factors [50,51]. Our data indicated that PCN could induce oxidative damage in U937 cells and antioxidant NAC inhibited PCN-induced IL-8 protein expression. In most cases, PCN’s cytotoxicity has been strongly linked to its potential effects on redox cycle. When entering into cells, PCN oxidizes intracellular pools of NADPH, NADH and GSH directly by accepting electrons, and it passes these electrons to oxygen leading to sustained generation of ROS (O_2_^_^ and H_2_O_2_) under aerobic condition [25]. Oxidative damage results in unbalance between the oxidant and antioxidant processes. Antioxidant defense system (enzymatic scavengers SOD, CAT and so on and some smal1 molecule antioxidants including NAC, GSH, vitamin C and vitamin E) plays an important role in the elimination of oxygen radical [52]. Cellular GSH levels have been reported to influence the activity of a number of transcription factors, including NF-κB, AP-1, and HIF-1α [53,54]. NAC is a thiol compound that has direct antioxidant properties and also is converted to GSH by cells and thereby limits oxidant-mediated cell injury. By demonstrating the inhibitory effect of NAC on PCN-induced IL-8 production, we indicate that NAC can act as a protective factor that mitigates PCN pro-inflammatory effect on differentiated U937 cells.

In short, in this study, we found that PCN could induce PMA-differentiated U937 cells to produce IL-8 by activating MAPKs and NF-κB signaling pathways. Our further studies will focus on understanding the interaction between p38 MAPK, ERK and other cytokine regulators. Knowledge of the mechanisms by which PCN induces PMA-differentiated U937 cells to produce cytokines may provide better understanding and rational approaches for the control of PCN-induced inflammatory processes.

## Conclusions

PCN induces U937 cells in a concentration- and time- dependent manner to increase IL-8 mRNA expression and secretion. Furthermore, MAPKs and NF-κΒ signaling pathways may be involved in the expression of IL-8 in PCN-exposed U937 cells, indicating that the green pus streptozotocin in the *P.aeruginosa* infection has an important role in inflammation reactions. PCN or TNF-α alone could induce PMA-differentiated U937 cells to express IL-8, but no synergistic effect was observed between these two factors. The mechanism requires further study.

## Competing interests

The authors declare that they have no competing interests.

## Authors’ contributions

WC made substantial contributions to conception and design of this work. JZ and YD drafted the manuscript. WL and YL revised it critically for important intellectual content. XY and WL were responsible for the experimental operation. DP carried out the analysis and interpretation of the data. BC made final approval of the version to be published. All authors read and approved the final manuscript.
